# Assessment of the nucleotide modifications in the high-resolution cryo-electron microscopy structure of the *Escherichia coli* 50S subunit

**DOI:** 10.1093/nar/gkaa037

**Published:** 2020-01-28

**Authors:** Vanja Stojković, Alexander G Myasnikov, Iris D Young, Adam Frost, James S Fraser, Danica Galonić Fujimori

**Affiliations:** 1 Department of Cellular and Molecular Pharmacology, University of California San Francisco, San Francisco, CA 94158, USA; 2 Department of Biochemistry and Biophysics, University of California San Francisco, San Francisco, CA 94158, USA; 3 Department of Bioengineering and Therapeutic Sciences, University of California San Francisco, San Francisco, CA 94158, USA; 4 Quantitative Biosciences Institute, University of California San Francisco, San Francisco, CA 94158, USA; 5 Department of Pharmaceutical Chemistry, University of California San Francisco, 600 16th St, MC2280 San Francisco, CA 94158, USA

## Abstract

Post-transcriptional ribosomal RNA (rRNA) modifications are present in all organisms, but their exact functional roles and positions are yet to be fully characterized. Modified nucleotides have been implicated in the stabilization of RNA structure and regulation of ribosome biogenesis and protein synthesis. In some instances, rRNA modifications can confer antibiotic resistance. High-resolution ribosome structures are thus necessary for precise determination of modified nucleotides’ positions, a task that has previously been accomplished by X-ray crystallography. Here, we present a cryo-electron microscopy (cryo-EM) structure of the *Escherichia coli* 50S subunit at an average resolution of 2.2 Å as an additional approach for mapping modification sites. Our structure confirms known modifications present in 23S rRNA and additionally allows for localization of Mg^2+^ ions and their coordinated water molecules. Using our cryo-EM structure as a testbed, we developed a program for assessment of cryo-EM map quality. This program can be easily used on any RNA-containing cryo-EM structure, and an associated Coot plugin allows for visualization of validated modifications, making it highly accessible.

## INTRODUCTION

Ribosomes are complex cellular machines responsible for protein synthesis. Accurate translation by the ribosome requires specific post-transcriptional and post-translational modifications of ribosomal RNA (rRNA) and ribosomal proteins, respectively. The roles of individual rRNA modifications are still poorly understood, and while some modulate the function of the ribosome, others are important in RNA folding and stability (1–4). The majority of rRNA modifications are located in functional regions of the ribosome: the decoding center, the peptidyl transferase center (PTC), the exit tunnel or the interface between ribosomal subunits. In *Escherichia coli*, not a single rRNA modification is essential for the survival of the cell. However, the presence of individual modifications confers advantages under certain growth or stress conditions (3). For instance, modifications of rRNA have emerged as one of the most clinically relevant mechanisms of resistance to ribosome-targeting antibiotics (5–8). Differential expression and post-translational modifications of ribosomal proteins, as well as differences in rRNA modifications, can lead to heterogeneity in ribosome composition which can result in ‘specialized ribosomes’. These ribosomes can have a substantial impact on the relative abundances of proteins being produced and consequently influence an organism's adaptability to a variety of environmental factors (9–11). For example, in bacteria, ribosome heterogeneity has been identified as a mechanism for stress adaptation (12).

Visualizing rRNA modifications is important for understanding their impact on the translating ribosome, and can aid in the development of novel antibiotics. Recently, rRNA and ribosomal protein modifications have been visualized in several high-resolution X-ray and cryo-electron microscopy (cryo-EM) structures of bacterial ribosomes (2.3 Å resolution) (13), including *E. coli* ribosome (2.4 Å) (14), parasitic (2.8 Å – cryo-EM) (15) and human ribosomes (2.8 Å – cryo-EM) (16,17). In certain instances, assignments of nucleotide modifications have been subsequently revised in the light of improved data (18), highlighting the challenges in assigning nucleotide modifications in near-atomic resolution structures.

Here, we report a high-resolution cryo-EM structure of the *E. coli* 50S subunit. Motivated by the high resolution of this map and previous claims in the literature about the ability to identify post-transcriptional modifications directly from the cryo-EM maps (17), we developed a program, qPTxM (quantifying Post-Transcriptional Modifications), to validate the presence of assigned rRNA modifications. These metrics provide a sensitive readout of map quality by probing the local density around all possible modifications of each nucleotide. Modifications that are well-supported by the map can be easily examined in Coot using a custom plugin (19). The performance of this program in confirming biochemically-validated modifications provides orthogonal evidence of the superior quality of this map relative to previous EM maps. Additionally, we corroborated the assigned nucleotide conformations using the X3DNA software system (20). Overall, these analyses confirm the quality of this new high-resolution cryo-EM map of the *E. coli* 50S ribosome and suggest that even higher quality maps are required before post-transcriptional modifications can be confidently identified *de novo* from cryo-EM data alone.

## MATERIALS AND METHODS

### *E. coli* 50S ribosomal subunit purification

50S ribosomal subunit was purified from *E. coli* MRE600 strain using modified version of previously published protocol (21). In short, cells were grown to an OD_600_ of 0.5 in LB media at 37°C and 220 rpm. Cells were pelleted by centrifugation, washed with wash buffer (20 mM Tris pH 7.5, 100 mM NaCl, 10 mM MgCl_2_) and stored at –80°C. The pellet corresponding to 750 ml of growth was resuspended in Buffer A (20 mM Tris pH 7.5, 300 mM NH_4_Cl, 10 mM MgCl_2_, 0.5 mM EDTA, 6 mM β-mercaptoethanol and 10 U/ml of SuperASE-In (Ambion)). PMSF was added to a final concentration of 0.1 mM. The cell resuspension was lysed using microfluidizer at 10 000 psi with three passages. The lysate was centrifuged at 16 100 rpm (Beckman Ti45 rotor) for 30 min, at 4°C, to remove cell debris. Without disturbing the pellet, supernatant was removed and centrifuged for additional 30 min. The resulting supernatant was loaded onto a 32% sucrose cushion prepared in Buffer A and centrifuged at 28 000 rpm (Beckman SW41Ti rotor) for 16 h, at 4°C, to obtain the crude 50S pellet. This pellet was slowly resuspended at 4°C in Buffer A to homogeneity. Any presence of non-resuspended particles was removed by a short centrifugation at 10 000 rpm, for 10 min at 4°C.

Crude 50S ribosomal subunits were further purified on 10–40% sucrose gradients. Sucrose gradients were prepared in Buffer A using BioComp Gradient Master. Approximately 650 pmol of crude 50S subunits were loaded on each gradient and centrifuged at 28 000 rpm (Beckman SW41Ti rotor) for 16 h, at 4°C. Fractions were collected from top to bottom using BioComp Piston Gradient Fractionator. The sample absorbance was recorded using UV reader and the peak corresponding to 50S was pooled for precipitation with PEG 20 000. A final concentration of 10.5% PEG 20 000 was slowly added to the pooled fractions, incubated on ice for 10 min and centrifuged at 10 000 rpm, for 10 min at 4°C. The pure 50S pellet was dissolved in Buffer A and filtered using 0.22 μm low-binding Durapore PVDF filters (Millipore).

### Cryo-EM analysis

**Figure 1.**
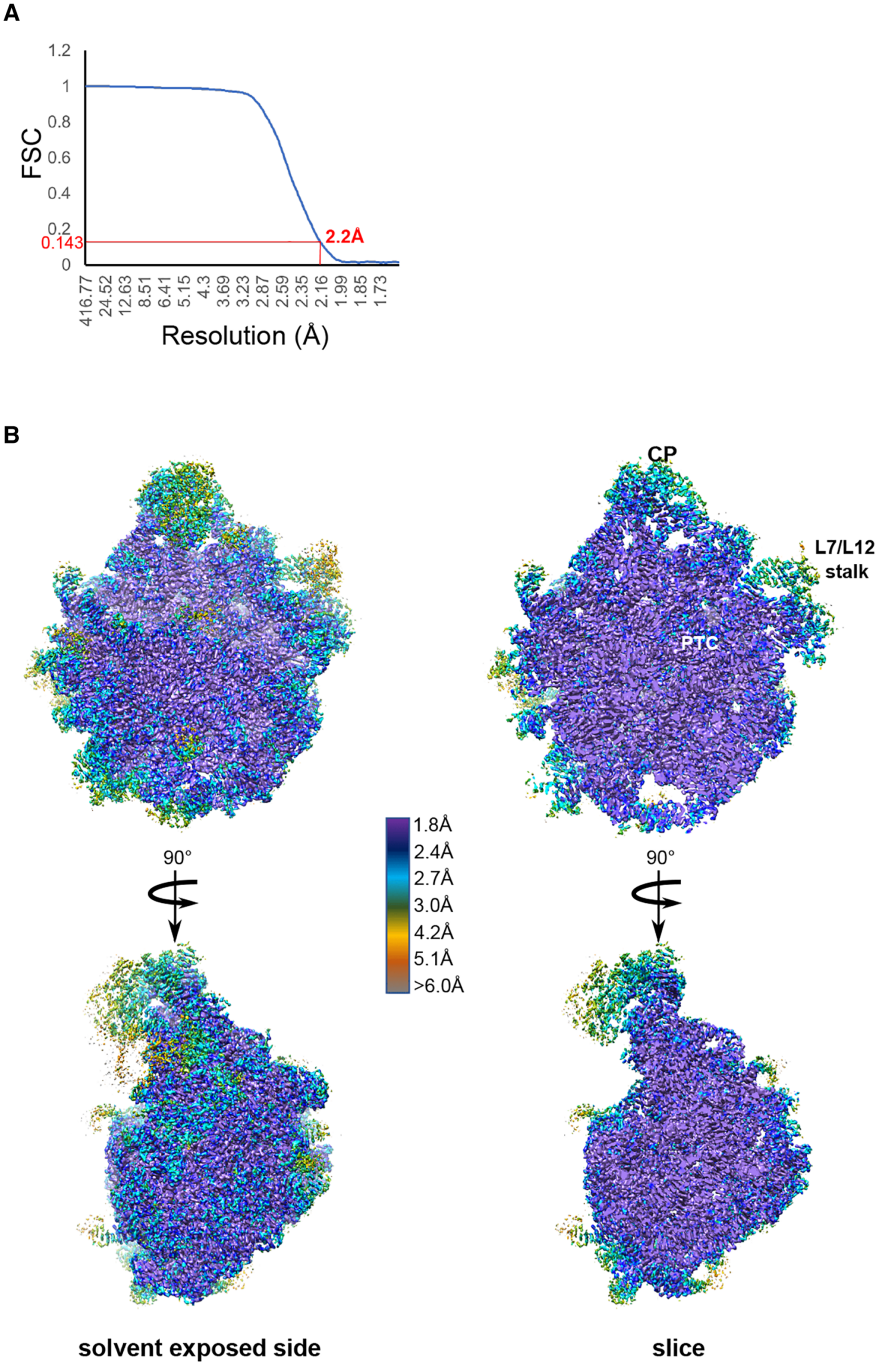
Cryo-EM data collection and processing. (**A**) Fourier shell correlation (FSC) curve indicating the overall resolution of the *E. coli* 50S subunit using the gold-standard criterion (FSC = 0.143). (**B**) 3D cryo-EM map of the 50S *E. coli* subunit colored according to local resolution. Local resolution map was calculated with ResMap. Left, solvent exposed side view; right, cut-through view. Features in the 50S subunit include the central protuberance (CP), peptidyl-transferase center (PTC), L7–L12 region (L7/12 stalk).

Purified 50S ribosomal subunits were diluted from 2 to 0.5 mg/ml in Buffer A, applied to 300-mesh carbon coated (2nm thickness) holey carbon Quantifoil 2/2 grids (Quantifoil Micro Tools) and flash-frozen as described in (22). Data were collected on the in-house Titan Krios X-FEG instrument (Thermo Fisher Scientific) operating at an acceleration voltage of 300 kV and a nominal underfocus of Δ*z* = 0.2 to 1.5 μm at a magnification of 29 000 (nominal pixel size of 0.822 Å). We recorded 1889 movies using a K2 direct electron detector camera in super-resolution mode with dose fractionation (80 individual frames were collected, starting from the first one). Total exposure time was 8 s, with the total dose of 80 e^-^ (or 1 e^-^/Å^2^/frame). Images in the stack were aligned using the whole-image motion correction and patch motion correction (5 × 5 patches) methods in MotionCor2 (23). Before image processing, all micrographs were checked for quality and 1610 best were selected for the next step of image processing. The contrast transfer function of each image was determined using GCTF (24) as a standalone program. For particle selection we have used Relion3.0 autopicking procedure which picked 193 000 particles (25). For the first steps of image processing we used data binned by a factor of 8 (C8 images). During the first round of 2D classification we removed only images with ice or other contaminants (162 746 particles left). Subsequently, the initial structure was generated using the *ab initio* procedure in CryoSPARC v2.0. Following this step, we performed Relion 3D classification with bin by four data (C4) in order to exclude bad particles (21 197 non-ribosomal particle images). The resulting 141 549 particle images of ribosomes were used for subsequent classification and refinement procedures. For the initial refinement we used spherical mask, which was followed by further refinement using mask around stable part of 50S (excluding L1 stalk, L7/L12 region). A further improved cryo-EM map was obtained by using CTF-refinement procedure from Relion 3.0. The Post-processing procedure implemented in Relion 3.0 (25) was applied to the final maps with appropriate masking, B-factor sharpening (automatic *B*-factor estimation was −55.85) and resolution estimation to avoid over-fitting (final resolution after post-processing with 50S mask applied was 2.68 Å). Subsequently the stack of CTF-refined particles was processed in a new version of CryoSPARC v2.0 (26). After homogeneous refinement resolution was improved to 2.5 Å. The same stack of particles was additionally refined in cisTEM (27). After Auto-Refine (with automasking within cisTEM) we attained 2.4 Å resolution. Then we performed local refinement using 50S mask (the same one used for refinement in Relion) and also applied per particle CTF refinement as implemented in cisTEM software. After such refinement the resolution was improved to 2.2 Å (Figure 1). This map after Sharpen3D (27) was used for model building and map interpretation.

### Atomic model building and refinement

The final model of the 50S subunit was generated by multiple rounds of model building in Coot (19) and subsequent refinement in PHENIX (28). Modified nucleotides were drawn and the restraints for the atomic model fitting and refinements were generated using JLigand (29). The atomic model of the 50S subunit from the *E. coli* ribosome structure (PDB 4YBB) (14) was used as a starting point and refined against the experimental cryo-EM map by iterative manual model building and restrained parameter-refinement protocol (real-space refinement, positional refinement, and simulated annealing). Final atomic model comprised ∼154 836 atoms (excluding hydrogens) across the 3016 nucleotides and 3356 amino acids of 29 ribosomal proteins. Proteins L3, L10 and L31 were not modelled in. In addition, 185 Mg^2+^, 3897 water molecules, one Zn^2+^ and one Na^+^ were included in the final model. Prior to running MolProbity (30) analysis, nucleotides 878–898, 1053–1107, 2099–2188 of 23S rRNA, and ribosomal proteins L9 and L11 were removed, due to their high degree of disorder. Overall, protein residues and nucleotides show well-refined geometrical parameters (Table 1). Figures were prepared using UCSF Chimera 1.13 (31).

**Table 1.** Cryo-EM data collection, refinement and validation statistics

**Data collection and processing**	
Electron microscope	Krios
Magnification	29 000
Number of micrographs	2688
Number of particles in the map	300 000
Number of particles after classification	144 000
Pixel size (Å)	0.822
Defocus range (μm)	–0.2 to –1.5
Voltage (kV)	300
Electron dose (e-/Å^2^)	80
**Map refinement**	
Model resolution (Å)	2.2
FSC threshold	0.143
Model resolution range (Å)	2.2–20
Map sharpening *B*-factor (Å^2^)	–70
**Refinement and model statistics**	
Clashscore, all atoms	4.98
Protein geometry	
MolProbity score	1.87
Rotamer outliers (%)	2.41
Cβ deviations >0.25 Å (%)	0.5
Ramachandran (%)	
- Favored	95.26
- Allowed	4.68
- Outliers	0.07
Deviations from ideal geometry	
- Bonds (%)	0.01
- Angles (%)	0.18
Nucleic acid geometry	
Probably wrong sugar puckers (%)	0.67
Bad backbone conformations (%)	14.15
Bad bonds (%)	0.05
Bad angles (%)	0.02

### qPTxM program for quantitative identification of post-transcriptional modifications: development and analysis

**Figure 2.**
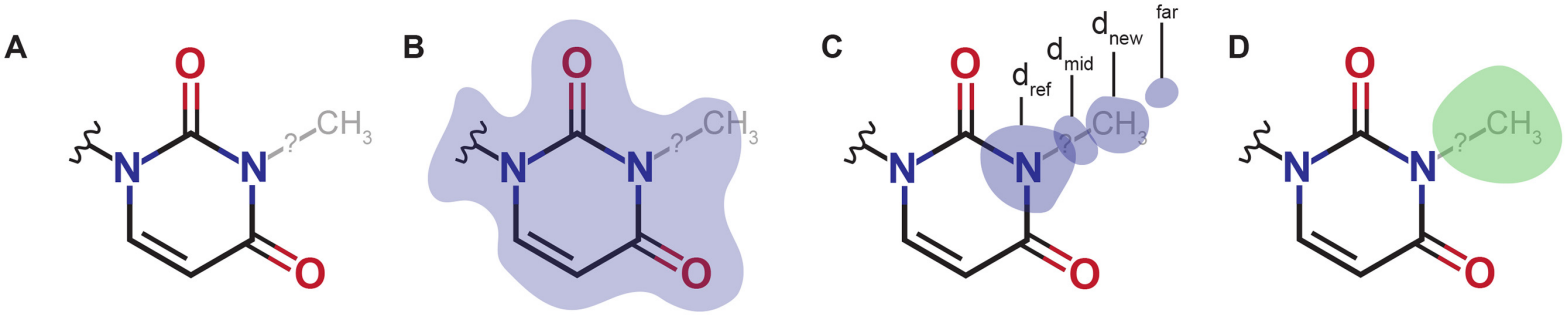
Methodology for assignment of post-transcriptional modifications (PTxMs). (**A**) For each possible modification, the new atoms are modeled with ideal geometry on a copy of the model. (**B**) Correlation of the model with the map is used to remove modifications for which the model is already a poor fit in the density. (**C**) Ratios of densities at several locations on the modified residue are used to assess whether the density profile along the vector of each new covalent bond matches expectations. By default, *d*_far_ may be no more than twice *d*_new_, *d*_far_ may not be more than d_mid_, and d_ref_ may be no more than three times *d*_new_. (**D**) A difference map is calculated between the experimental map and a map calculated from the (unmodified) model, and only the modifications with the strongest experimental and difference map densities at the new atom positions (by default, the 50% strongest by each measure) are considered. An illustration of these parameters is shown for N3-methyluridine.

qPTxM was developed for validation and detection of post-transcriptional modifications based on evidence from an X-ray or EM map. It requires a map, an atomic model refined into it, and the estimated average resolution of the map. First, qPTxM reads the model and constructs a copy containing only the major conformer of each residue or nucleotide, removes all hydrogens, and removes any post-transcriptional modifications already modeled. The resulting model is used to generate a calculated map. Using the calculated and experimental maps, a difference map is generated, where positive difference density features will indicate sites of possible unmodeled atoms. qPTxM then examines each nucleotide and corresponding density in the experimental and difference maps for possible modifications—this excludes pseudouridines, which differ from uridines only in the attachment of the base to the ribose (Figure 2, Supplementary Figure S1). First, the correlation coefficient between the calculated and experimental maps is calculated using 8-point interpolation at the position of each atom in the unmodified nucleotide. If the correlation coefficient is worse than 0.6, the quality of the density is considered insufficient to support the presence of modifications, and no modifications will be suggested at this nucleotide. The default threshold of 0.6 is a lower bound that may be adjusted upward for higher confidence and lower sensitivity where high resolution data permit more stringent criteria.

Second, densities at four groups of positions in the experimental map are calculated and compared. For each modification we define one or more reference atoms (ref), one or more new atoms (new), positions midway between the reference and the new atoms (mid), and positions half a covalent bond beyond the new atoms along the direction of the bond (far). We obtain the experimental map densities at each position by 8-point interpolation and calculate the average density within each of these categories. Ratios of these densities *d*_ref_, *d*_mid_, *d*_new_ and *d*_far_ are used as indications of how closely the shape of the density around the modification matches our expectation, and we specifically select three comparisons that help eliminate a large number of cases where features in the map at the proposed modification position can be explained by water molecules, ions, and protrusions from other parts of the protein or nucleic acid model. We have found that requiring *d*_far_ ≤ 1.5*d*_new_, *d*_far_ ≤ *d*_mid_ and *d*_ref_ ≤ 3*d*_new_ filters out most of these cases. These multipliers may also be adjusted with optional arguments to qPTxM.

Third, the collection of possible modifications is sorted by *d*_ref_ and *d*_new_diff_, where the latter quantity is the density measured by 8-point interpolation at the new atom positions in the difference map, and the modifications in the lower half of *d*_ref_ and the lower 90% of *d*_new_diff_ densities are excluded from consideration. This step improves selectivity for nucleotides that have strong densities and strong evidence for additional unmodeled atoms. At all stages, proposed modifications that are marked as rejected are not removed from memory, so that all filters operate on the full set of proposed modifications. The full set is written to all_tested_ptms.out to facilitate rapid filter-only runs of qPTxM, while ptms.out contains all accepted modifications.

Finally, modifications are scored by taking the ratio *d*_new_/*d*_ref_ and inflating it by a factor between 1.2 for atoms close to the aromatic ring (in modifications such as 7-methylguanosine (m^7^G)) and 3 for atoms distant from the aromatic ring (in modifications such as 2-methylguanosine (m^2^G)). Thus, the score is normalized depending on how far the modified position is from the aromatic ring and whether the reference atoms are part of the aromatic ring or protrude from it. This scaling factor helps account for the steeper dropoff in density at positions further from other heavy atoms. Scores are compared against a default threshold of 0.5, which can again be adjusted with an optional argument.

Modifications passing all tests are built into a copy of the model for visualization, with the possibility of multiple modified residues at a single position, which is written to a file ptms.pdb. qPTxM also generates a plain text file ptms.out, a script goto_ptms.py and pdfs of histograms of the correlation coefficients, scores and densities. It is possible to rerun just the filtering steps of qPTxM to regenerate plots and output (.out) files for rapid fine-tuning of the optional parameters using the argument adjust_filters_only=True, since these analyses can be run on the ptms.out file alone without repeating each of the calculations. This can be useful for adjusting sensitivity until a reasonable number of modifications pass all tests. To generate the custom script and the copy of the model with modifications in place, qPTxM should be run again without this argument once the user has settled on final parameters. The command ‘coot goto_ptms.py pruned.pdb ptms.pdb’ will load two copies of the model in the Coot model viewing program, where pruned.pdb contains no modifications and ptms.pdb contains those passing all tests (Supplementary Figure S2). The script goto_ptms.py adds functionality to jump directly to each modification for visual inspection.

We used synthetic data to tune the behavior of qPTxM under ideal (noise-free) conditions. We are including the tools we used to generate the synthetic data in qPTxM for the benefit of other users and developers, accessed by supplying the command line argument synthetic_data=True. Beginning with a refined map and model, we strip off any modifications already present, any alternate conformers, and all hydrogens, just as for a model to be tested. This model is then modified at random at 10% of all recognized nucleotides (i.e. all standard, unmodified nucleotides as well as any modified nucleotides the program recognizes how to strip modifications off of). Then, instead of using the experimental map supplied, the modified model is used to generate calculated structure factors (substituting for a noise-free experimental map) and the experimental map supplied is used only to inform the dimensions of the calculated map. Subsequently, qPTxM proceeds through all other steps as described above, and finishes by reporting on its accuracy. The list of (true) modified sites in the synthetic data is also written to a file synthetic_ptms.out and can be examined independently.

We primarily intended detection of post-transcriptional modifications to be useful as a validation step when the modifications are already known by some other method. The performance of the program should improve with the quality of the map, which should track with its resemblance to a noise-free synthetic map. To this end, we trained a random forest classifier on features from synthetic data generated at a range of resolutions (2, 2.2, 2.4, 2.6, 2.8, 3.2, 3.8 and 5 Å) and a range of B-factors (10–90 in increments of 10) from a collection of 22 publicly available ribosome models after stripping any recognized modifications and then modifying 10% of nucleotides at random. For datasets 6EK0 and 6QZP, the deposited map and model did not match, so the map was adjusted to 0.85 Å voxel size from 0.9 Å and fit with Chimera to a map calculated from the model. Features were the same map densities, ratios and correlation coefficients used to filter proposed modification sites, the scores determined by qPTxM, and the resolution and B-factors used to generate the synthetic maps. The trained classifier has an out-of-bag score estimate of 99.5% and was able to identify detectable (non-pseudouridine) modifications in the present dataset with a mean accuracy score of 99.8%. The classifier can be used to predict a set of modifications by running a separate script, rf_util.py, with python3 (a requirement for loading the classifier object). The output from this script can be fed back to qPTxM with the selected_ptms argument to produce the matching model for visualization if desired. We used the classifier to predict modifications for the same 22 ribosomes in addition to the current dataset and an additional group of synthetic datasets generated at 2.2 Å resolution and a B-factor of 10. Even with >99% accuracy, few true positives could be detected in most experimental maps. Both sensitivity and specificity were markedly better for the noise-free synthetic datasets compared with the experimental datasets (Supplementary Figure S3, Supplementary Tables S1 and S2), validating our hypothesis that unambiguously identifiable modifications in EM maps may be used as evidence of high map quality. The classifier and script to use it are available as part of the qPTxM github repository (https://github.com/fraser-lab/qptm). The data used to train the classifier and scripts reproducing these steps are also available there. Curious developers are highly encouraged to reproduce and improve upon our classifier and any other aspect of the implementation.

## RESULTS

Using a Titan Krios X-FEG instrument equipped with a K2 direct electron detector camera operated in super-resolution mode and performing reconstruction refinement in Relion3, CryoSPARC and cisTEM software, we obtained a structure of the *E. coli* 50S subunit at an average resolution of 2.2 Å (Table 1 and Figure 1). In order to avoid any model bias, the initial *ab initio* structure was generated in CryoSPARC and then used as a reference in Relion3. To achieve higher resolution, later steps of 3D refinement included only the well-ordered regions of the 50S subunit, and excluded the L1 stalk and L7/L12 region. An improved cryo-EM map was subsequently obtained using the CTF-refinement function of Relion3 and further improved using per-particle CTF refinement in cisTEM (see Methods). The resulting local resolution map is uniformly sampled, with a large portion of the map showing resolution of ∼2 Å (Figure 1B). Highly dynamic peripheral regions of the 50S subunit, encompassing the L1 arm, the stalk proteins L10, L11 and L7/L12 region, ribosomal proteins L9 and L31, and sections of the GTPase center, however, are disordered. While proteins L1, L7, L10 and L31 are not built in our model, the amino-terminal domains of the proteins L9 and L11 are structurally well-defined and thus these two proteins are built into the final model. Conformations and complete models of proteins L9 and L11 were modeled based on the high-resolution X-ray crystal structure (14). Overall, our structure is the highest resolution cryo-EM ribosome structure to date, allowing us to unambiguously visualize many rRNA modifications, Mg^2+^ ions and coordinated waters.

### Ribosomal modifications

**Figure 3.**
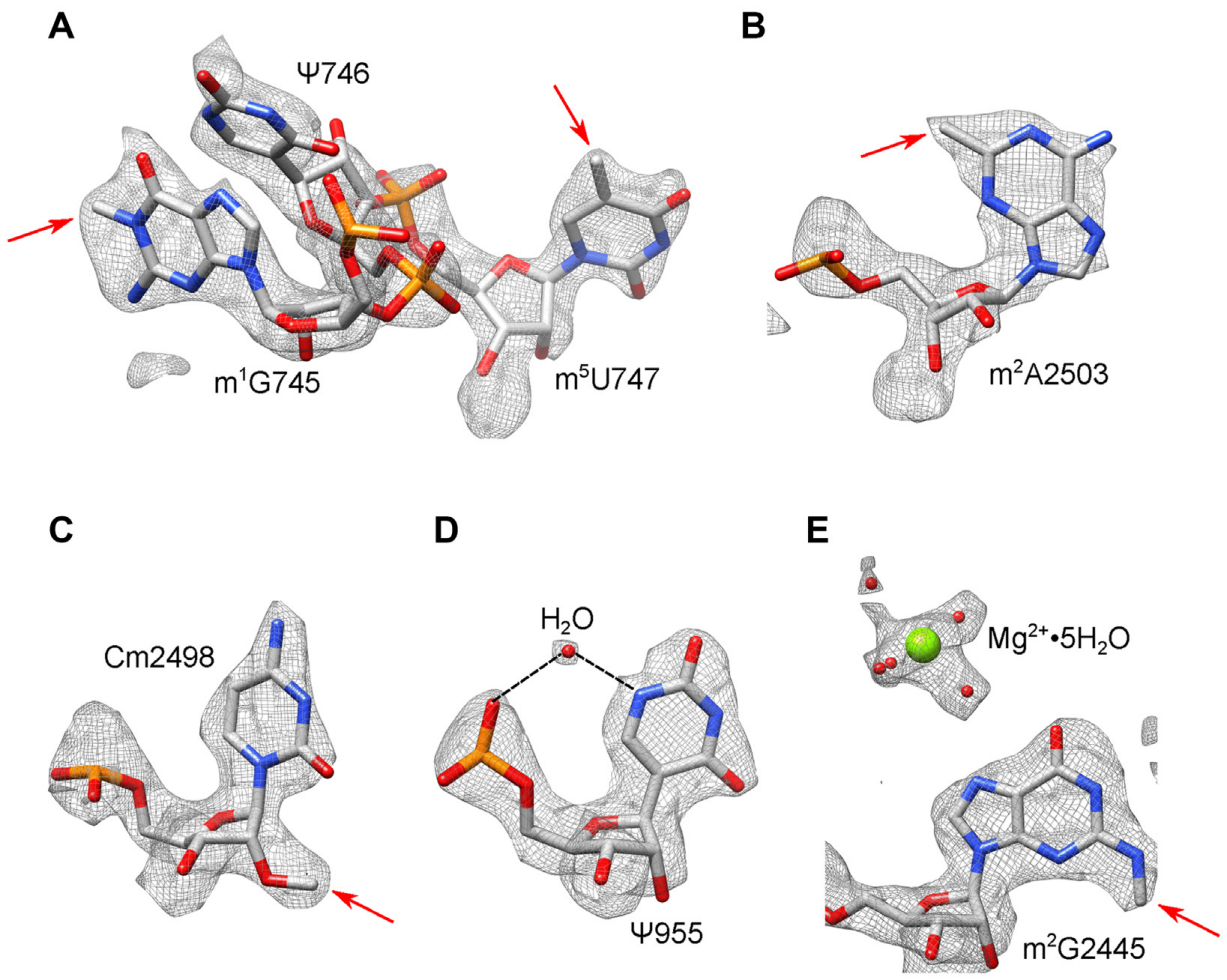
Cryo-EM density map allows modeling of rRNA modifications of the *E. coli* 50S ribosomal subunit. (A–D) Cryo-EM density map of selected modified nucleotides present in 23S rRNA. The modifications shown are: (**A**) 1-methylguanosine (m^1^G) 745, pseudouridine (Ψ) 746, 5-methyluridine (m^5^U) 747, (**B**) 2-methyladenosine (m^2^A) 2503, (**C**) 2′-O-methylcytidine (Cm) 2498, (**D**) pseudouridine (Ψ) 955 and (**E**) 2-methylguanosine (m^2^G) 2445. (D, E) Coordinated waters and Mg^2+^ are also shown.

Most ribosomal modifications in the 50S subunit are clustered in the PTC region and the nascent peptide exit tunnel (NPET). Excluding pseudouridines (Ψ), we modeled 23 out of 25 known post-transcriptional modifications in the 23S rRNA, including the non-planar base of dihydrouridine (D) at position 2449 (Figure 3, Supplementary Figure S4 and Supplementary Table S3). The only modifications not observed are substoichiometric thionation of cytidine 2501 (s^2^C) and disordered methylated pseudouridine 1915 (m^3^Ψ1915). Nucleotide 1915 is located at the interface between the large and the small ribosomal subunits and it has been proposed to strengthen the intersubunit contact (13). Seven out of ten known pseudouridines were well-ordered and thus are modelled in our structure (Supplementary Figure S4). Unlike uridines, pseudouridines contain an extra hydrogen-bond donor in the form of an N1 imino group, and water-mediated contact between the pseudouridine N1 imino group and the rRNA phosphate backbone is taken as an indication of pseudouridine presence. This water-mediated contact was observed for several pseudouridines (Supplementary Figure S4 and Supplementary Table S4).

### Validating map quality by detection of ribosomal modifications: qPTxM

The observation that post-transcriptional modifications were discernible in this map presented us with an opportunity to use these features to benchmark map quality. Others have noted (e.g. (17)) that the sites of modifications can potentially be discovered from the map directly. We reasoned that the success rate of recovering the correct modification positions could serve as a proxy for overall map quality. To test this hypothesis, we developed a new program, qPTxM (for quantifying post-transcriptional modifications). The premise of qPTxM is similar to EMRinger (32), which quantifies agreement between the protein backbone and EM density maps. In both programs, the part of the structure that can be most confidently built (the nucleobase or the protein backbone) is used to assess the strength of the EM density support at positions that are predicted, with ideal geometry, to be occupied by features that require high resolution data to model accurately (the nucleotide modification or the gamma atom of the protein side chain). These approaches leverage prior knowledge of ideal geometry to objectively quantify the strength of density at positions where a model might be extended, in this case identifying possible sites of post-transcriptional modification. qPTxM reports on true positives, false positives, false negatives, and true negatives. It can alternatively be used for modification discovery in very high resolution maps: qPTxM produces a ranked list of candidate sites with support for interactive viewing in Coot (19) (Supplementary Figure S2) (see Materials and Methods), but is subject to a high false-positive rate.

To test for modifications on an EM model and map, we examine the evidence for each possible modification on each nucleotide and apply a series of filters to progressively narrow the pool of candidates. We optimized these filters for noise-free, synthetic maps we generated for this purpose, achieving sensitivity >50% and specificity >99% in all synthetic test datasets (see Materials and Methods and Supplementary Figure S3). First, where the local map quality at a given nucleotide is insufficient for interpretation, all possible modifications at this nucleotide are rejected (3542 out of 11 712 possible modifications in the ribosome model and experimental EM map considered here). Second, we test whether there is density supporting a new atom at the appropriate position, and whether a covalent bond is more likely than a nearby water molecule or ion (7459 out of 8260 remaining modifications are rejected in this step). Third, we select for the most well-resolved reference atoms and the modification positions with the strongest difference density, rejecting another 600 of the remaining 801 modifications. Finally, we produce a score for each modification, and all 201 remaining modifications pass a final filter on score.

qPTxM was able to identify 8 of 13 modifications theoretically visible in the density. This represents a higher sensitivity than we observed for the other six ribosome cryoEM datasets that we tested, and even with 193 false positives, a comparable specificity to the other structures (Supplementary Figure S3). Although our 8 true positives include some of the highest scoring candidates (Supplementary Table S5), many of the other top candidates are residues that have never been detected as modified in *E. coli* 23S rRNA by sensitive mass spectrometry methods (33). This result suggests that although the map quality is high enough to confirm known modified residues and to guide their refinement, it is not yet sufficient to unambiguously determine the modification state of bases. Thus, a major caveat in automatic map interpretation by qPTxM is a high false positive rate, an attribute that is likely shared by a blinded, manual interpretation of the density map.

To attempt to address the high false positive rate, we trained a random forest classifier on a collection of synthetic data generated from publicly available structures and tested its performance on a separate group of synthetic maps (Supplementary Figure S3, Supplementary Table S2). Predictions by the classifier exhibited higher sensitivity (higher true positive rate) but lower specificity (higher false positive rate) than modifications identified by qPTxM with default parameters. Results were comparable by both methods (and more widely variable generally) for the experimental maps, with the present dataset outperforming most others in both cases (Supplementary Figure S3).

Because qPTxM searches for modifications using only the information from a map and unmodified atomic model, a second application of the program for modification discovery is also possible. When examining a high-resolution structure without prior knowledge about the modifications on any of the nucleotides, qPTxM can alternatively reduce the labor required to search for possible modifications by supplying the user with a limited number of candidate sites to be investigated by biochemical methods. The researcher's independent examination of the candidate sites in Coot may still identify a number of cases where features in the density can be otherwise explained. In addition to reducing the number of sites to examine by a factor of ∼50, qPTxM applies criteria uniformly to all nucleotides, eliminating any confirmation bias favoring sites that appear modified when the map is contoured to a particular level (Supplementary Figure S5).

### Nucleotide conformations in the ribosome

RNA nucleobases can adopt two major conformations, *syn* and *anti*, that differ in the position of the base with respect to the sugar ring, and are interchangeable through rotation around the glycosidic bond. While the *anti* conformation is energetically more favorable than the *syn* conformation, the *syn* state affords a more compact form of the nucleotide aiding in the stability of RNA (34,35). Detailed analysis of several RNAs and RNA-containing complexes including ribosomes indicated that most *syn* nucleobases participate in tertiary stacking and tertiary base-pairing (34). Consequently, this conformation often occurs in the functionally important regions of RNA, where *syn* pyrimidines are rarer than *syn* purines, as pyrimidine derivatives have a larger energetic penalty to adopting this conformation. The importance of *syn* conformations to RNA function is still not completely understood, and high-resolution structures are instrumental in identifying conserved *syn* nucleotides and aiding in assessment of their functional role.

**Figure 4.**
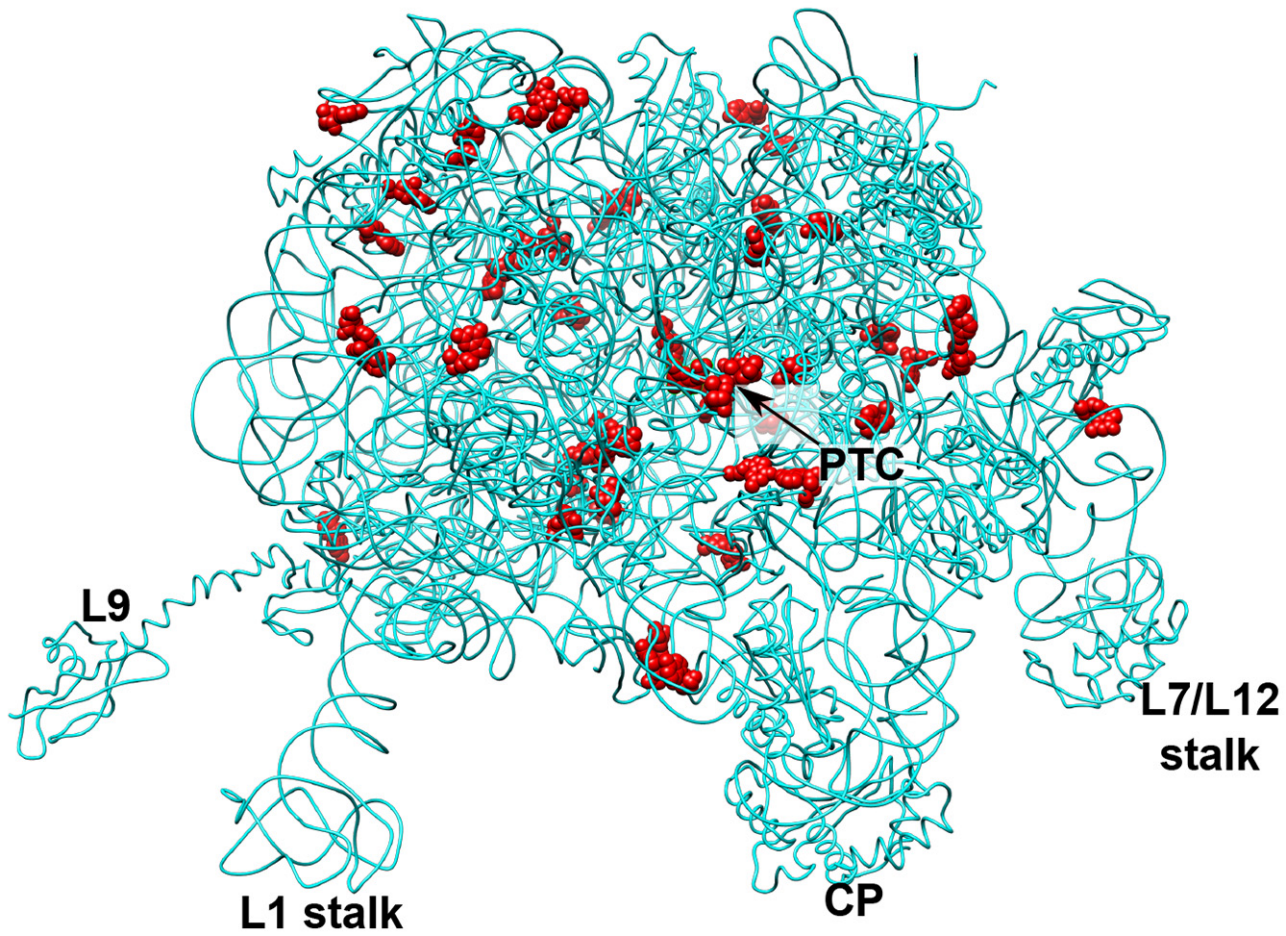
*Syn* nucleotides with good density present in cryo-EM structure are shown as red spheres. *Syn* conformation is defined by glycosidic torsion angle (χ) of 0° ± 110°. Features in the 50S subunit include the central protuberance (CP), L1 arm (L1 stalk), protein L9 (L9), L7–L12 region (L7/12 stalk) and the peptidyl transferase center (PTC).

Glycosidic torsion angle (χ) for each nucleotide present in our structure was measured using X3DNA suite, an integrated software system for analysis of nucleic-acid containing structures (20). The *syn* conformation was defined with a χ-angle in a range of 0° ± 110°, following recommendations from recent analysis of high-resolution RNA datasets (36). In the 23S rRNA, we observed 43 *syn* purines and five *syn* pyrimidine with good densities (Figure 4, Supplementary Table S6 and Supplementary Table S7). Assigned *syn* nucleotides were compared to *syn* bases reported in other high resolution *E. coli* ribosome structures, such as the one presented in (14) (Supplementary Table S6 and Supplementary Table S7). We observed high similarity between the two structures, suggesting that differences in experimental conditions between two techniques had minimal effect on the conformations of the nucleotides, and that designated *syn* nucleotides most likely have an important structural or functional role (34). In our structure, *syn* nucleotides are observed throughout the 23S rRNA (Figure 4), however when examining each domain, the largest number of *syn* nucleotides are found in the domain V of 23S rRNA, whose central loop lines the PTC and NPET (Figure 4, Supplementary Table S6 and Supplementary Table S7). One example is highly conserved nucleotide G2576, which occupies *syn* conformation extending the stacking between G2576 and G2505. This stacking supports the single stranded rRNA segment of the central loop of domain V, which lines the NPET and is a binding site for several antibiotics (Supplementary Figure S6A). Additionally, in the PTC region, nucleotide A2503 methylated at the C2 position (m^2^A) also adopts the full *syn* conformation. It has been proposed that the presence of this methylated nucleotide in the *syn* conformation extends the stacking between A2059 and A2503, stabilizing the fold of the two single-stranded rRNA sections (13). *Syn* nucleotides also support tertiary structure through tertiary base-pairing. For example, in the helix H20 of domain I, A330 assumes the *syn* conformation to form *trans* Watson–Crick/sugar edge A-G base pair (37) with G307, a nucleotide part of the helix H19 (Supplementary Figure S6B). This base pairing stabilizes the interaction between two helices, an interaction which is additionally facilitated by the ribosomal protein L24. Protein L24 is an assembly initiator protein and organizer of rRNA folding (38).

### Solvation of the PTC and NPET

Most clinically relevant antibiotics that target the 50S subunit bind in the PTC or in the NPET region. These antibiotics inhibit protein synthesis by either perturbing the binding of tRNA at the A- or P-sites or by preventing the passage of the nascent peptide chain through the ribosomal tunnel (7). In the previous X-ray crystal structures, ordered water molecules were observed in the known antibiotic-binding sites, including PTC and NPET (14). High-resolution of the new cryo-EM structure allowed for the modelling of water molecules which were assigned using a utility supplied within Coot (19). However, while water molecules coordinating magnesium ions were evident and well-resolved throughout the structure (Supplementary Figure S7), only a few ordered water molecules were observed in the PTC and NPET. We hypothesize that water molecules and ions are diffused throughout the NPET, and could become ordered in the presence of the nascent peptide or an antibiotic.

## DISCUSSION

In the current study, we have harnessed several technological advances to obtain the highest resolution cryo-EM ribosome structure to date at an average resolution of 2.2 Å. This resolution allowed for the modelling of known RNA modifications. Additionally, we present a new program for assessing map quality, named qPTxM, which demonstrates the superior quality of the present map.

It is now possible to resolve small features such as post-transcriptional modifications in sufficiently high-resolution cryo-EM maps. For structures where modifications have been determined by biochemical methods, such as the *E. coli* ribosome, we can use our ability to rediscover these features in the map as a proxy for map quality. This is the design principle guiding development of qPTxM. Because program execution involves rediscovering these modifications, it can also be used for *de novo* discovery of modifications in a map of sufficiently high quality. We caution against using cryoEM as the sole discovery method for assignment of post-transcriptional modifications, as recent work has shown the potential for false identification of modifications (17,18). Consistent with that emerging view, our analyses indicate that it is not yet possible to identify true modifications without also producing many false positives, although we anticipate that additional methodological advancements in cryo-EM will soon make this viable. We therefore describe this alternative use of qPTxM in advance of a map suitable for this use of the software.

The present structure can be used for comparison to other ribosome structures. While ribosomal RNA is extensively modified across all three kingdoms of life, the nature and the location of the rRNA modifications differ between kingdoms. For example, C1962 is a 5-methylcytidine (m^5^C) in *E. coli* and *Thermus thermophilus* 23S rRNA, and unmethylated in archaea and eukaryotes (39). Presence of a methyl group on C1962 extends the stacking between G1935–C1962 within the H70–H71 helical junction. As helix H71 participates in the formation of bridge B3 with 16S rRNA, it is possible that C1942 aids in strengthening of the intersubunit contact and subsequently in maintaining the 70S stability (40). Differences in the extent of modification at this nucleotide could indicate dissimilarities in the optimization of intersubunit contact and 70S stability at distinct temperatures and in different environments.

Another interesting discovery from comparison of our structure to other ribosome structures is the extent of solvation of functional regions, such as the NPET. Water molecules are known to play both functional and structural roles, especially in charged molecules such as nucleic acids. While in our structure the water molecules appear to be disordered throughout the NPET, in the high-resolution X-ray structure several well-ordered waters are identified (14). We hypothesize that these differences in the hydration pattern between the two structures are real (not a limitation of the resolution) and likely due to differences in experimental methods. One possibility is that rigid packing in the crystal lattice and dehydration procedures prior to cryocooling likely promotes ordering of the water in the X-ray structure. Alternatively, quicker vitrification during preparation of the cryo-EM sample might have aided in capturing a more biologically relevant state (41). Ultimately, understanding the hydration pattern in the ribosome structure will be important for future computational studies, calculations of the electrostatic contribution of water molecules (42) and other ions (43), and might aid in the development of new antibiotics that target the bacterial ribosome.

Lastly, our structure also allowed us to determine the conformation of each nucleotide in the structure. We have identified 48 *syn* nucleotides, predominantly in the functionally important regions of the ribosome, where they likely have a functional or structural role. Our findings are highly consistent with previous *syn* designations in X-ray crystal structure (14) and previous analysis that revealed the overabundance of *syn* nucleotides in the antibiotic-binding sites (44). *Syn* conformation has been proposed to be associated with the higher flexibility of rRNA in these sites, resulting in pockets that are capable of the induced fit-binding mode. Thus, the identification of the *syn* nucleotides in the high-resolution structures, and especially *syn* nucleotides conserved across kingdoms could facilitate the identification of new functional sites suitable for the design of ribosome targeting drugs.

## DATA AVAILABILITY

Atomic coordinates have been deposited in the Protein Data Bank under accession number 6PJ6, the density map has been deposited in the EMDB under accession number 20353.

## Supplementary Material

gkaa037_Supplemental_File
