# Supplementary material for: Assessment of the nucleotide modifications in the high-resolution cryo-electron microscopy structure of the Escherichia coli 50S subunit
Source: Nucleic Acids Res. 2020 Jan 28;48(5):2723–32. doi: 10.1093/nar/gkaa037 (PMC7049716; doi:10.1093/nar/gkaa037)
Supplement: gkaa037_Supplemental_File [file gkaa037_supplemental_file.pdf]

SUPPLEMENTARY INFORMATION

High-resolution cryo-electron microscopy of the *Escherichia coli* 50S subunit and validation of nucleotide modifications

Vanja Stojković[1], Alexander G. Myasnikov[2], Iris D. Young[3], Adam Frost[2,4*], James S. Fraser[3,4*], Danica Galonić Fujimori[1,4,5*]

SUPPLEMENTARY FIGURES AND TABLES LEGENDS

Supplementary Figure S1. Diagram of the series of tunable filters applied to candidate modifications by qPTxM. Individual filters select the modification sites where map density or some related measurement most closely matches expectations. Examples of the filters and their associated measurements are shown at right for a candidate N3-methylation on a uridine. Two filters, difference densities fraction and reference densities fraction, are determined as the thresholds selecting particular proportions of the possible modifications, while other filters are encoded as absolute thresholds. All thresholds or proportions, including the score cutoff, can be adjusted by passing optional command line arguments to qPTxM. The accepted modifications are those that pass all the filters, as represented by the funnel.

Supplementary Figure S2. A custom plugin script goto_ptms.py is written out with each run of qptm.py. Supplying this script to Coot on launch along with the original and modified models allows the user to step through all suggested sites of post-transcriptional modifications.

Supplementary Figure S3. Plot of qPTxM results for the test ribosome maps in receiver operating characteristic (ROS) space. True positive rate is calculated as the ratio of the number of true positives to the sum of the true positives and false negatives. False positive rate is calculated as the ratio of the number of false positives to the sum of the false positives and true negatives. An inset at top right calls attention to the sub-5% false positive rate across all datasets. Four groups of datasets are plotted, corresponding to the two groups of datasets (either the maps and models available in the PDB, or noise-free synthetic maps generated from these models with a random 10% of sites modified) and the two ways of assessing candidate modifications (either by running qPTxM with all default parameters except resolution or by predicting modifications with a random forest classifier). The random forest classifier was trained on a collection of synthetic maps from the same models with a different random 10% of sites modified in each instance, and was not trained on any of the data plotted or on any map generated from the model presented here. True positive rates could not be calculated for experimental maps with no true positives, so experimental points are shown only for structures with known modifications. We added any modifications known to be present in *E. coli*, *S. cerevisiae* or *H. sapiens* to ribosome structures in these organisms where not already modelled.

Supplementary Figure S4. Cryo-EM density map of modified nucleotides in the 50S subunit.

Supplementary Figure S5. Visual similarity of modified and unmodified positions in the present map (A) and a 2.9 Å resolution human ribosome map, PDB 6EK0 (B). The danger of manual visual analysis of maps and propensity for confirmation bias is demonstrated by comparing the site of a true positive to three sites of false positives with similar density for each map. Maps are contoured at 5 sigma in all cases.

Supplementary Figure S6. Example of *syn* nucleotides participating in tertiary base stacking (A) and tertiary base pairing (B). (A) Nucleotide G2576 in 23S rRNA adopts a *syn* conformation that extends the stacking between G2576 and G2505. G2505 lines the peptide exit tunnel and interacts directly with the nascent peptide. Model of VemP nascent peptide chain (PDB 5NWY) (1) is superimposed on the *E. coli* 50S structure. (B) Nucleotide A330 assumes the *syn* conformation to form trans Watson-Crick/sugar edge A-G base pair with G307. G307 and A330 are part of the two loops in helices H19 and H20, respectively. These loops are additionally stabilized through interaction with ribosomal protein L24 (cyan color). Hydrogen bonds are indicated by black dashed lines.

Supplementary Figure S7. An example of water coordinated to magnesium ions. Phosphate of a nucleotide U567 in 23S rRNA also coordinates shown magnesium ion.

Supplementary Table S1. Comparison of the qPTxM default behavior and random forest predictions on the 22 publicly available ribosomes tested and the present dataset. Where modifications were known to be present from other structures from the same organism, but not modeled in a particular model, these modifications were added to the model and contribute to true positives and false negatives. The reported resolution in the Protein Data Bank was passed as the only non-default parameter to qPTxM.

Supplementary Table S2. Comparison of the qPTxM default behavior and random forest predictions on synthetic datasets generated from the 22 publicly available ribosomes tested and the present dataset. Where modifications were known to be present from other structures from the same organism, but not modeled in a particular model, these modifications were added to the model and contribute to true positives and false negatives. Reported resolutions of the original models (presumably affecting model geometries) is listed, but all synthetic maps were generated at 2.2 Å resolution and with all residues reset to a B-factor of 10.

Supplementary Table S3. Modified nucleotides in the *E. coli* 50S subunit.

Supplementary Table S4. Solvation of pseudouridines.

Supplementary Table S5. List of nucleotides known to be modified in *E. coli* 50S subunit with their qPTM scoring percentile. Scores for the assigned modifications were determined using a calculated cryo-EM map at a 2.2 Å resolution. Scores were calculated on nucleotides whose correlation coefficients between experimental and calculated cryo-EM maps were at least 0.6. Modifications were limited to the 60% of nucleotides that had the strongest experimental map density at reference atom positions and the 10% of sites with the strongest difference map density at the positions of modifications. Modifications were also filtered by three thresholds on the ratios of densities in the experimental map, $d_{far} \leq 0.6 \cdot d_{new}$, $d_{far} \leq 0.4 \cdot d_{mid}$ and $d_{ref} \leq 3.5 \cdot d_{new}$ (see Methods). Scores are scaled ratios of difference and experimental map densities at the proposed and reference atom positions, respectively, and percentiles were calculated among the 176 sites passing all tests and scoring at least 0.5. NP indicates that the modification did not meet these conditions and was not assigned a score. For the nucleotide 1915, methylation was tested starting from a modelled uridine, as pseudouridine cannot be identified from the map alone.

Supplementary Table S6. Comparison of *syn* purines with good density between cryo-EM structure and high-resolution X-ray crystal structure (2). *Syn* conformation is defined by glycosidic torsion angle (χ) of 0° ± 110°. *Syn* purines present only in high-resolution X-ray crystal structure are shown in bold.

Supplementary Table S7. Comparison of *syn* pyrimidines with good density between cryo-EM structure and high-resolution X-ray crystal structure (2). *Syn* conformation is defined by glycosidic torsion angle (**χ**) of 0° ± 110°. *Syn* pyrimidines present only in high-resolution X-ray crystal structure are shown in bold.

REFERENCES

1. Su, T., Cheng, J., Sohmen, D., Hedman, R., Berninghausen, O., von Heijne, G., Wilson, D.N., Beckmann, R. (2017) The force-sensing peptide VemP employs extreme compaction and secondary structure formation to induce ribosomal stalling. *Elife*. **6**, e25642.
2. Noeske,J., Wasserman,M.R., Terry,D.S., Altman,R.B., Blanchard,S.C. and Cate,J.H.D. (2015) High-resolution structure of the Escherichia coli ribosome. *Nat. Struct. Mol. Biol.*, **22**, 336–341.

Supplementary Figure S1.

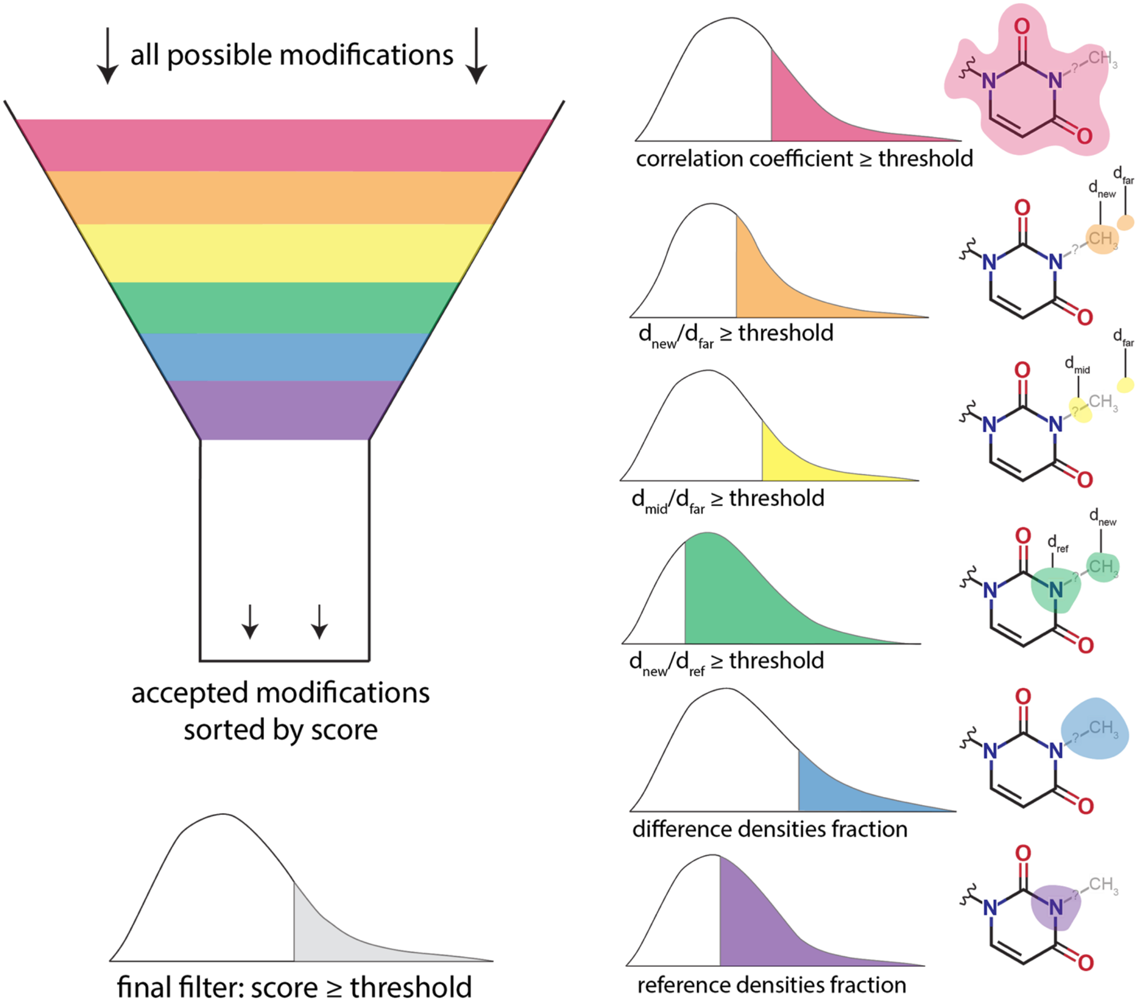

Supplementary Figure S2.

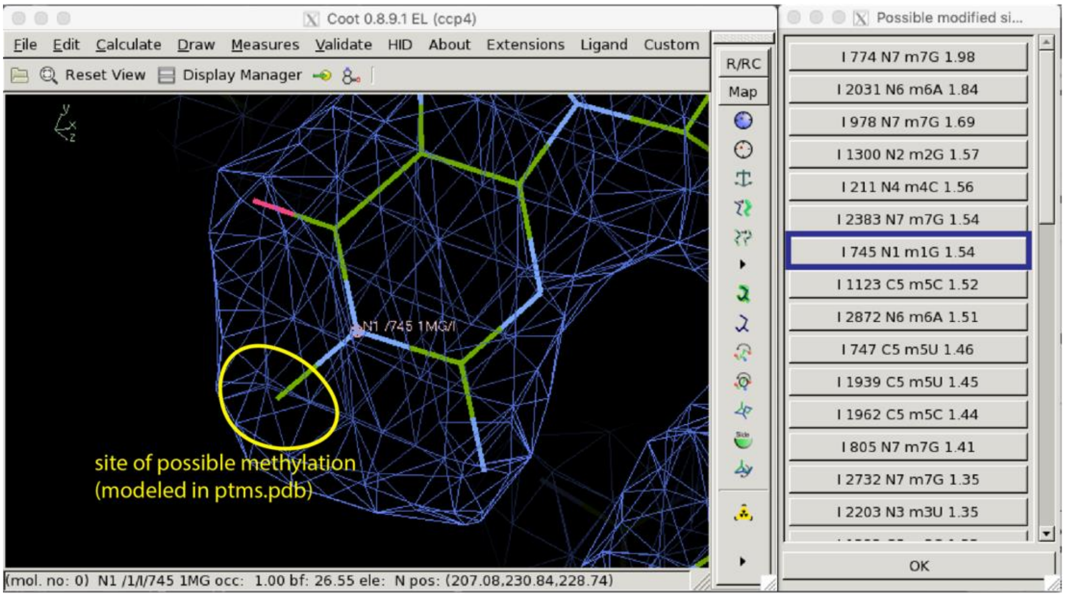

Supplementary Figure S3.

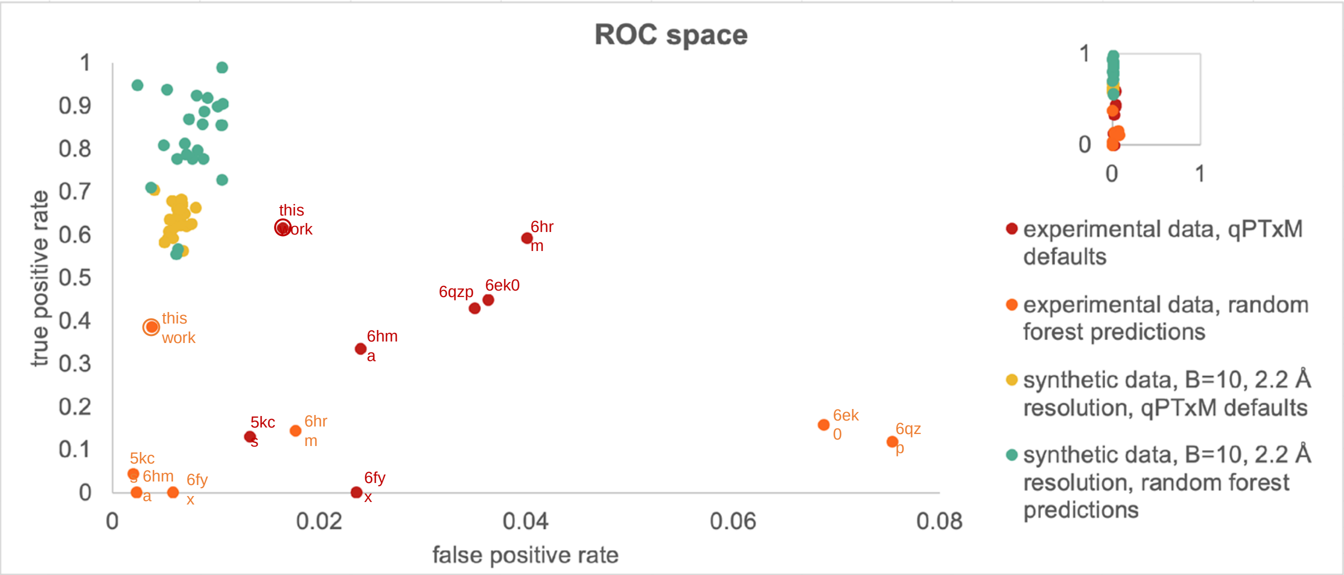

Supplementary Figure S4.

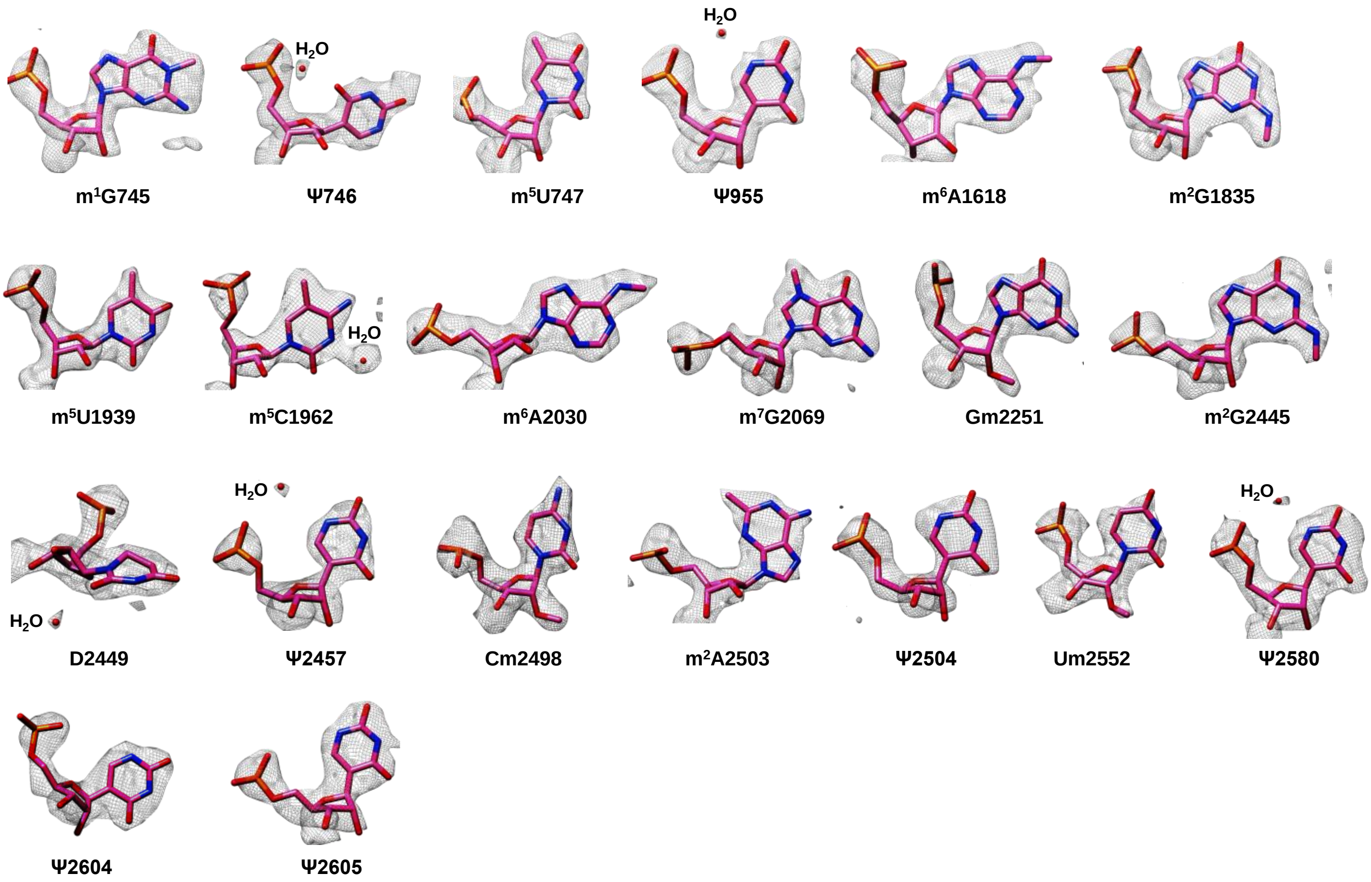

Supplementary Figure S5.

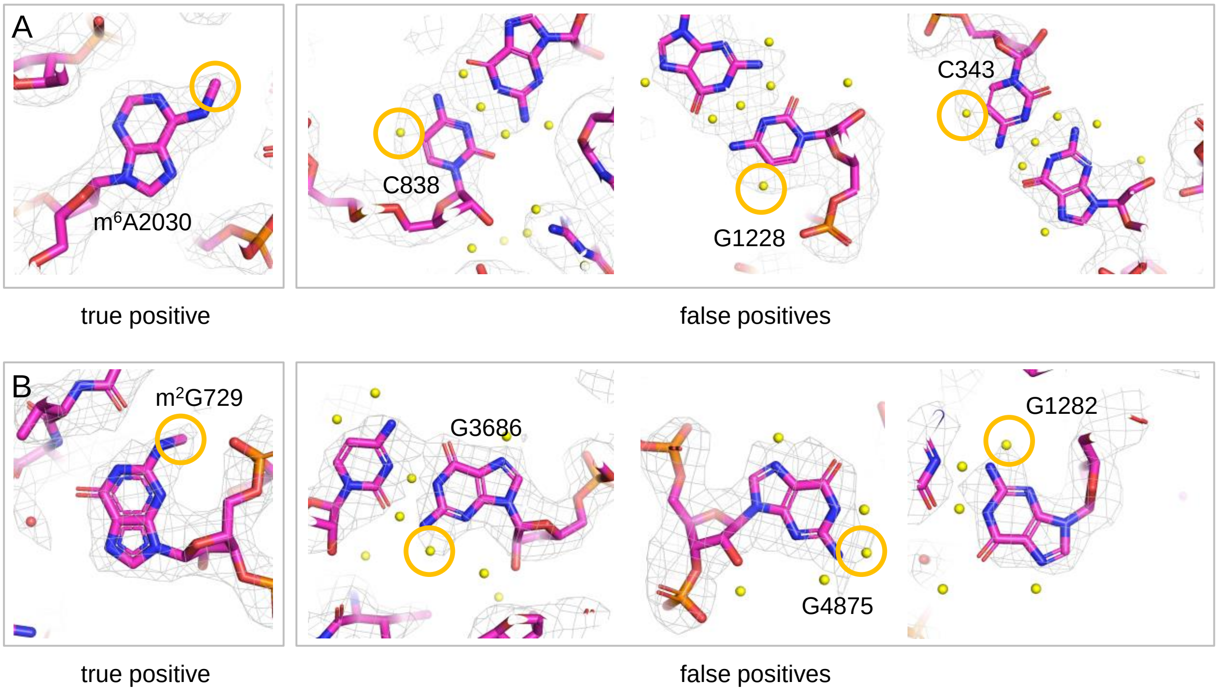

Supplementary Figure S6.

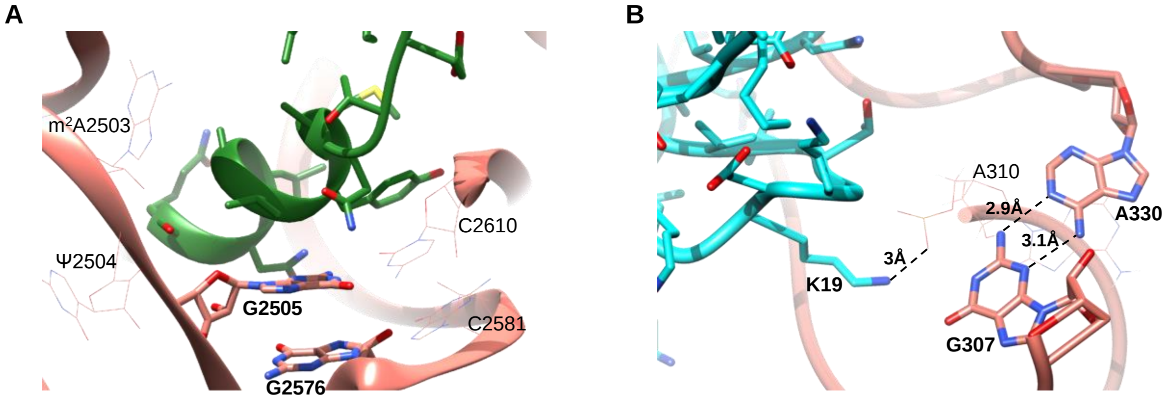

Supplementary Figure S7.

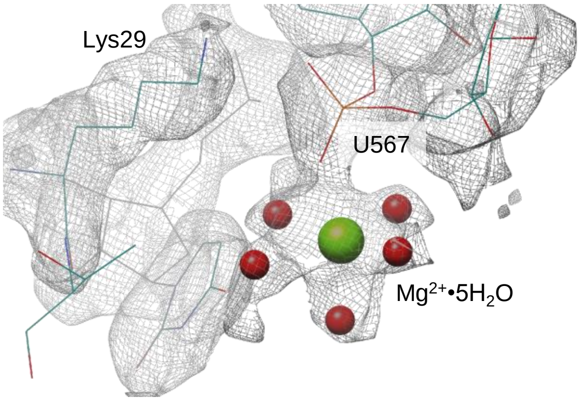

Supplementary Table S1.

				experimental data qPTxM default parameters				experimental data random forest predictions			
**model**	**subunit**	**organism**	**dmin (Å)**	true +	false +	false -	true -	true +	false +	false -	true -
5kcs	70S	*E. coli*	3.9	3	239	20	17733	1	37	22	17935
5zeb	70S	*M. smegmatis*	3.4	0	422	0	18322	0	750	0	17994
6dzi	70S	*M. smegmatis*	3.46	0	505	0	18025	0	1380	0	17150
6dzk	30S	*M. smegmatis*	3.6	0	143	0	5760	0	398	0	5505
6dzp	50S	*M. smegmatis*	3.42	0	331	0	12296	0	1416	0	11211
6ek0	80S	*H. sapiens*	2.9	34	803	42	21280	12	1519	64	20564
6fyx	48S	*S. cerevisiae*	3.5	0	166	10	6865	0	41	10	6990
6gaw	55S	*S. scrofa*	3.2	0	244	0	9655	0	112	0	9787
6gaz	28S	*S. scrofa*	3.1	0	103	0	3786	0	444	0	3445
6gb2	39S	*S. scrofa*	3.2	0	134	0	6136	0	462	0	5808
6gq1	80S	*S. cerevisiae*	4.4	0	458	0	19983	0	908	0	19533
6gxm	70S	*E. coli*	3.8	0	320	0	17718	0	469	0	17569
6gz3	80S	*S. cerevisiae*	3.6	0	455	0	21787	0	69	0	22173
6gzq	70S	*T. thermophila*	3.28	0	409	0	17488	0	1305	0	16592
6h4n	100S	*E. coli*	3	0	184	0	17839	0	567	0	17456
6ha1	70S	*B. subtilis*	3.1	0	512	0	17475	0	107	0	17880
6hiv	mt-LSSU	*T. brucei*	7.8	0	52	0	4331	0	98	0	4285
6hiw	mt-SSU	*T. brucei*	3.37	0	52	0	2196	0	188	0	2060
6hix	mt-LSU	*T. brucei*	3.39	0	66	0	2069	0	297	0	1838
6hma	50S	*S. aureus*	2.65	1	274	2	11129	0	27	3	11376
6hrm	70S	*E. coli*	2.96	13	711	9	17007	3	314	18	17404
6qzp	80S	*H. sapiens*	2.9	33	778	44	21426	9	1674	68	20530
this work	50S	*E. coli*	2.2	8	193	5	11506	5	45	8	11654

Supplementary Table S2.

				synthetic data qPTxM default parameters				synthetic data random forest predictions			
**model**	**subunit**	**organism**	**dmin (Å)**	true +	false +	false -	true -	true +	false +	false -	true -
5kcs	70S	*E. coli*	3.9	283	96	183	17433	376	87	90	17442
5zeb	70S	*M. smegmatis*	3.4	317	102	208	18117	408	141	117	18078
6dzi	70S	*M. smegmatis*	3.46	323	146	165	17896	276	114	212	17928
6dzk	30S	*M. smegmatis*	3.6	92	38	54	5719	125	50	21	5707
6dzp	50S	*M. smegmatis*	3.42	227	81	139	12180	284	108	82	12153
6ek0	80S	*H. sapiens*	2.9	337	125	215	21482	306	133	246	21474
6fyx	48S	*S. cerevisiae*	3.5	110	40	76	6815	151	48	35	6807
6gaw	55S	*S. scrofa*	3.2	171	65	84	9579	229	98	26	9546
6gaz	28S	*S. scrofa*	3.1	65	22	31	3771	90	20	6	3773
6gb2	39S	*S. scrofa*	3.2	105	41	49	6075	112	65	42	6051
6gq1	80S	*S. cerevisiae*	4.4	353	140	192	19756	423	124	122	19772
6gxm	70S	*E. coli*	3.8	265	120	206	17447	403	186	68	17381
6gz3	80S	*S. cerevisiae*	3.6	358	155	220	21509	410	81	168	21583
6gzq	70S	*T. thermophila*	3.28	297	133	178	17289	429	186	46	17236
6h4n	100S	*E. coli*	3	260	94	179	17490	345	126	94	17458
6ha1	70S	*B. subtilis*	3.1	286	105	177	17419	396	184	67	17340
6hiv	mt-LSSU	*T. brucei*	7.8	80	27	44	4232	110	38	14	4221
6hiw	mt-SSU	*T. brucei*	3.37	38	9	16	2185	43	18	11	2176
6hix	mt-LSU	*T. brucei*	3.39	37	13	19	2066	53	5	3	2074
6hma	50S	*S. aureus*	2.65	185	60	128	11033	289	90	24	11003
6hrm	70S	*E. coli*	2.96	281	113	146	17200	422	184	5	17129
6qzp	80S	*H. sapiens*	2.9	345	120	199	21617	499	201	45	21536
this work	50S	*E. coli*	2.2	160	58	115	11379	239	85	36	11352

Supplementary Table S3.

Position	Known modification	Cryo EM density
745	m^1G	+
746	Ψ	+
747	m^5U	+
955	Ψ	+
1618	m^6A	+
1835	m^2G	+
1911	Ψ	-
1915	$m^3Ψ$	- (a)
1917	Ψ	-
1939	m^5U	+
1962	m^5C	+
2030	m^6A	+
2069	m^7G	+
2251	Gm	+
2445	m^2G	+
2449	D	+
2457	Ψ	+
2498	Cm	+
2501	s^2C	- (b)
2503	m^2A	+
2504	Ψ	+
2552	Um	+ (c)
2580	Ψ	+
2604	Ψ	+
2605	Ψ	+

(a) disordered region
(b) partial modification
(c) poor density for modification

Supplementary Table S4.

**Pseudouridine**	***Synlanti* conformation**	**H_2O-binding to N1**	**H_2O mediated contact to**
746	*syn*	no	N/A
955	*anti*	yes	OP1 955<br>OP2 954
1911	*N/A*	N/A	N/A
1915	*N/A*	N/A	N/A
1917	*N/A*	N/A	N/A
2457	*anti*	yes	OP2 2456<br>OP2 2457
2504	*anti*	no	-
2580	*anti*	yes	OP2 2580
2604	*anti*	no	-
2605	*anti*	no	-

N/A = not applicable; no electron density present

Supplementary Table S5.

Nucleotide	Modification	Scoring percentile
745	m^1G	0.86
747	m^5U	NP
1618	m^6A	0.57
1835	m^2G	1.00
1915	$m^3\Psi$**	NP
1939	m^5U	0.43
1962	m^5C	NP
2030	m^6A	NP
2069	m^7G	0.14
2251	Gm	NP
2445	m^2G	0.71
2498	Cm	0.29
2503	m^2A	0.00
2552	Um	NP

Supplementary Table S6.

23S rRNA					
**Domain I**	**Domain II**	**Domain III**	**Domain IV**	**Domain V**	**Domain VI**
A 49	G 620	G 1288	A 1669	G 2238	G 2645
A 71	G 729	A 1301	G 1695	G 2250	G 2751
A 74	A 783	G 1311	G 1929	A 2267	A 2765
A 101	A 845	G 1332	A 1936	G 2286	G 2825
G 177	A 933	G 1452		A 2287	A 2879
A 196	A 984	A 1490		A 2430	
A 330	A 1021			A 2503	
**A 404**	A 1142			A 2518	
A 528	G 1210			G 2576	
A 532				G 2581	

Supplementary Table S7.

23S rRNA	
Nucleotide	Sugar pucker
C 323	C2'-endo
Ψ 746	C1'-exo
U 1313	C4'-exo
**U 1758**	C2'-endo
U 1779	C4'-exo
**U 2689**	C2'-endo
U 2884	C2'-endo
